# LHCA4 residues surrounding red chlorophylls allow for fine-tuning of the spectral region for photosynthesis in *Arabidopsis thaliana*

**DOI:** 10.3389/fpls.2022.1118189

**Published:** 2023-01-17

**Authors:** Xiuxiu Li, Lixia Zhu, Jince Song, Wenda Wang, Tingyun Kuang, Gongxian Yang, Chenyang Hao, Xiaochun Qin

**Affiliations:** ^1^ School of Chemistry and Chemical Engineering, University of Jinan, Jinan, China; ^2^ School of Biological Science and Technology, University of Jinan, Jinan, China; ^3^ Photosynthesis Research Center, Key Laboratory of Photobiology, Institute of Botany, Chinese Academy of Sciences, Beijing, China

**Keywords:** photosystem I, LHCI, LHCA4, red Chl, red shift, blue shift

## Abstract

Improving far-red light utilization could be an approach to increasing crop production under suboptimal conditions. In land plants, only a small part of far-red light can be used for photosynthesis, which is captured by the antenna proteins LHCAs of photosystem I (PSI) through the chlorophyll (Chl) pair *a*603 and *a*609. However, it is unknown how the energy level of Chls *a*603–*a*609 is fine-tuned by the local protein environment *in vivo*. In this study, we investigated how changing the amino acid ligand for Chl *a*603 in LHCA4, the most red-shifted LHCA in *Arabidopsis thaliana*, or one amino acid near Chl *a*609, affected the energy level of the resulting PSI-LHCI complexes *in situ* and *in vitro*. Substitutions of the Chl *a*603 ligand N99 caused a blue shift in fluorescence emission, whereas the E146Q substitution near Chl *a*609 expanded the emission range to the red. Purified PSI-LHCI complexes with N99 substitutions exhibited the same fluorescence emission maxima as their respective transgenic lines, while the extent of red shift in purified PSI-LHCI with the E146Q substitution was weaker than in the corresponding transgenic lines. We propose that substituting amino acids surrounding red Chls can tune their energy level higher or lower *in vivo*, while shifting the absorption spectrum more to the red could prove more difficult than shifting to the blue end of the spectrum. Here, we report the first *in vivo* exploration of changing the local protein environment on the energy level of the red Chls, providing new clues for engineering red/blue-shifted crops.

## Introduction

Oxygenic photosynthesis uses light energy to convert carbon dioxide and water into carbohydrates and oxygen, sustaining almost all life activities on Earth. Photosynthesis is initiated with the light reactions, whereby light energy is captured and transferred to induce the charge separation in the reaction centers of photosystem I (PSI) and photosystem II (PSII), leading to the generation of reducing power as NADPH and metabolic energy as ATP. In vascular plants, PSI and PSII have a common general organization including a core complex moiety (PSI core or PSII core) and an outer antenna complex moiety (LHCI for PSI or LHCII for PSII). These two super-complexes are thus called PSI-LHCI and PSII-LHCII. Notably, their spectroscopic properties are substantially different: PSI can use light at wavelengths longer than 700 nm, whereas PSII cannot, even though they both use chlorophyll *a* (Chl *a*) as their major light-harvesting pigment (Croce and van Amerongen, 2014). This red shift in the absorption forms of PSI-LHCI expand the range of light-harvesting capacity into the near far-red region of the light spectrum, which is important for at least two major aspects. First, because far-red light is enriched in shaded environments, these red forms are helpful in harvesting light within or under a canopy (Rivadossi et al., 1999; Chen and Blankenship, 2011). Second, these red-shifted Chls greatly affect the excitation energy transfer within PSI-LHCI due to their low energy (Croce and van Amerongen, 2013). Therefore, a better understanding of the mechanism of these red forms of PSI-LHCI associated with Chl *a* is important for tuning the spectral region and regulating the excitation energy transfer of photosystems.

In vascular plants, the red spectral forms of PSI-LHCI are associated with a small number of low-energy Chl molecules (so-called red Chls) that absorb photons at wavelengths longer than 700 nm (Croce and van Amerongen, 2013). These red Chls bound to different sites of PSI-LHCI can have different energy levels that can be determined by fluorescence emission spectroscopy at low temperature (77K fluorescence). The PSI core complex contains 14 subunits (PsaA–L, PsaN, and PsaO) and 98 Chl *a* molecule (with six belonging to the electron transfer chain cofactors, while the other 92 function as antenna pigments) (Mazor et al., 2015; Qin et al., 2015) and shows a major fluorescence emission peak at 720 nm rather than two peaks at 685 nm and 695 nm as seen with PSII due to the existence of several red Chls (Satoh, 1986; Bassi and Simpson, 1987; Qin et al., 2006). The outer antenna complex associated with PSI (LHCI) is composed of four antenna proteins, LHCA1 to LHCA4, which are organized in the order LHCA1-LHCA4-LHCA2-LHCA3 from PsaG to PsaK, forming a crescent shape surrounding the PSI core on the PsaG–PsaF–PsaJ–PsaK side (Ben-Shem et al., 2003) and enhancing the PSI absorption cross section. These LHCA antenna proteins belong to the light-harvesting chlorophyll *a*/*b* protein superfamily (LHCs), which also includes the antenna proteins of PSII (LHCBs) (Buchel, 2015). Different LHC proteins share a similar fold, characterized by three major transmembrane helices (TMHs), B, C, and A, from their N- to C-terminal ends, and have conserved Chl- and carotenoid-binding sites (Qin et al., 2015). However, the most striking feature of LHCAs when compared to LHCBs is the presence of red Chl molecules in LHCAs (Croce and van Amerongen, 2013). These red Chls are critical for the fluorescence properties of LHCAs: While LHCBs have an absorption maximum in the range of 660–680 nm and an emission maximum at about 685 nm (Nussberger et al., 1994), the incorporation of the four LHCAs into PSI confers PSI-LHCI with an emission peak at about 735 nm, thus representing a red shift of about 50 nm compared to LHCB (Croce, 2015). Many studies have explored the mechanistic basis of these energy levels and the origin of the red Chl forms in individual LHCAs.

Work focusing on the spectral characteristics of the red Chls in LHCAs can be divided into two stages. In the 1980s, LHCI was often isolated as two fractions: LHCI-730 (the LHCA1-4 heterodimer) and LHCI-680 (a monomer or heterodimer composed of LHCA2 and LHCA3), named according to their fluorescence emission maxima (Kuang et al., 1984; Knoetzel et al., 1992). However, it was later found that a native LHCA2-LHCA3 heterodimer showed red-shifted fluorescence emission with a maximum around 730 nm, suggesting that LHCI-680 may not reflect the true native state of the heterodimer, as evidenced by the loss of the red shift, perhaps as a consequence of treatment with detergents during isolation (Wientjes and Croce, 2011). As several pigments are involved in the interaction between adjacent LHCAs and between LHCI and the PSI core (Qin et al., 2015), it was impossible to separate individual LHCAs without pigment loss (Ballottari et al., 2004), which prompted the use of reconstituted LHCA proteins with pigments (rLHCAs) for further study near the end of the 1990s. The four rLHCAs, rLHCA1–4, showed discrepancies in their fluorescence emission properties (Schmid et al., 1997; Schmid et al., 2002; Castelletti et al., 2003): rLHCA1 and rLHCA2 showed peaks at 686 and 701 nm, respectively, while rLHCA3 and rLHCA4 exhibited red-shifted peaks at 725 and 730 nm, respectively, demonstrating that 1) the energy level of the red Chls differed in different rLHCAs and 2) that rLHC4 had the most red-shifted absorption form.

It is important to reveal how the energy level of red Chls is regulated. Two excitonically coupled Chl *a* molecules are bound to the Chl-binding sites of A603 and B609 (nomenclature as described by Liu et al., 2004), corresponding to A5 and B5 according to Kühlbrandt et al. (1994), and were shown to be responsible for the red forms by means of site-directed mutagenesis of each rLHCA (Morosinotto et al., 2002; Morosinotto et al., 2003). We previously solved the crystal structure of the PSI-LHCI complex from garden pea (*Pisum sativum*) at 2.8-Å resolution in 2015 and recently improved the initial structure to 2.4-Å resolution (Qin et al., 2015; Wang et al., 2021). The PSI-LHCI structure illustrated how each LHCA binds to a pair of Chls (Chl *a*603– *a*609), which were suggested to be red Chls. The pair is the only Chl forming a connection between each LHCA and the PSI core at the stromal side, they occupy key positions in the excitation energy transfer (EET) pathway. Coupled with the lower energy level of red Chls, 90% of the energy absorbed by LHCI goes through the Chl *a*603-Chl *a*609 pair (Croce et al., 1996) and needs to be transferred across a high energy state to reach PSI core and be used for the primary reaction of PSI (Croce and van Amerongen, 2013). At the luminal pigment layer, in LHCA1, LHCA2, and LHCA4, a Glu residue near Chl *a*609 and located within helix C interacts with the C7-formyl group of Chl *b*607 *via* hydrogen bonds, while Chl *b*607 is the closest Chl molecule to Chl *a*603. In LHCA3 and LHCA4 with the most red-shifted emissions, the central ligand for Chl *a*603 is an Asn residue, while the central ligand for Chl *a*603 is a His residue in LHCA1 and LHCA2, which have weakly red-shifted emissions. By contrast, Chl *a*609 always coordinates with a Glu residue. In addition, based on pigment-pigment and pigment-protein interactions it was suggested that the porphyrin head of Chl *a*609 is more stable geometrically than Chl *a*603, and Chl *a*603 may be more likely to undergo conformational modulation. In spite of the high-resolution structures and some *in vitro* studies based on monomeric rLHCAs (Morosinotto et al., 2003; Wientjes et al., 2012), whether and how the protein matrix affects the energy level of the lowest energy state *in vivo* have not been reported.

In this study, we selected LHCA4, whose reconstituted form possesses the most red-shifted fluorescence emission among all rLHCAs, as the target protein, and focused on the effect of replacing the Asn(N)99 residue coordinating with Chl *a*603 and the Glu(E)146 residue located near Chl *a*609 to investigate the energy level of intact and mutant pigment-protein complexes *in vivo* (**Figure S1**). Accordingly, we generated a series of transgenic lines in Arabidopsis expressing constructs encoding single-point mutations in LHCA4 and determined their physiological and biochemical characteristics. In addition, we isolated and analyzed PSI-LHCI particles from the wild type and transgenic lines. We demonstrate here that all substitutions of the N99 residue in LHCA4 led to a blue shift in the fluorescence emission, indicative of the disruption of the red forms, while the E146Q mutation caused a shift red in the fluorescence emission, indicating an enhancement of the red shift. This is the first report of mutations in LHCA to be characterized *in vivo* in an effort to analyze the effects of protein micro-environments on the energy level of red Chls, which provides a new perspective of the regulation of these red Chl forms *in vivo*.

## Materials and methods

### Plant material and growth conditions

The Arabidopsis (*Arabidopsis thaliana*) accession Columbia-0 (Col-0) was used for all experiments. Plants (the wild type and mutants) were grown for 45 days in a growth chamber in 45% relative humidity, at 21°C, with a photoperiod of 16 h light/8 h dark under 110–130 μmol photons m^–2^ s^–1^. The environment was strictly controlled. For all measurements, only fully expanded mature leaves were used from the 4th to the 7th pair of leaves depending on the time and condition.

The *lhca4* mutant was prepared by CRISPR/Cas9-mediated gene editing, and the vector used was pHSE401 (Xing et al., 2014). The plant vector used to introduce transgenes with point mutations was pMDC83 harboring the cauliflower mosaic virus (CaMV) 35S promoter. Single nucleotide mutations were introduced by overlapping PCR. The sequence of the Arabidopsis genome was obtained from the TAIR website (https://www.arabidopsis.org/). Plasmids with the correct sequence were transformed into Agrobacterium (*Agrobacterium tumefaciens*) (strain GV3101), which were then employed for plant transformation. Transgenic plants were obtained after Agrobacterium infection by floral dipping, and lines homozygous for each transgene were identified in the T2 generation. See **Table S1** for a list of all primers.

### Thylakoid isolation and PSI-LHCI purification

Thylakoid isolation from Arabidopsis leaves was performed according to a previous report (Croce et al., 1996) with some modifications. Thylakoid membranes were prepared immediately after plant leaves were harvested. For every 10 g of leaves, 100 mL of solution A (20 mM Tricine-NaOH pH 7.8, 0.3 M sucrose, 5 mM MgCl_2_) was added and the tissues were crushed with a blender. The resulting cell and chloroplast suspension was then centrifuged at 7,000 × g for 7 min at 4°C. The supernatant was discarded, and the green pellet was resuspended in solution B (20 mM Tricine-NaOH, pH 7.8, 5 mM MgCl_2_) and then centrifuged at 20,000 × g for 10 min to obtain the thylakoid membrane in a JA14 rotor (Beckman) at 4°C. The chlorophyll concentrations were calculated from the absorbances at 645 and 663 nm of an 80% (v/v) acetone extract (Arnon, 1949).

PSI-LHCI purification was performed according to a published report (Ballottari et al., 2004). The thylakoid membrane was solubilized 1% (v/v) n-dodecyl β-D-maltoside (β-DDM) at 0.5 mg Chl/mL in an ice bath for 30 min. The insolubilized materials were removed by centrifugation at 40,000 × g for 15 min, and the supernatant was loaded onto a 0.3 M–0.9 M continuous sucrose density gradient (containing 20 mM Tricine-Tris, pH 7.5, 0.015% β-DDM) and centrifuged at 243,500 × g for 16 h at 4°C in an SW40Ti rotor (Beckman).

### SDS-PAGE and immunoblot analyses

The polypeptide compositions of samples were analyzed by SDS–PAGE. Samples were treated with a sample buffer containing 2% (w/v) lithium dodecyl sulfate, 60mM dithiothreitol and 60mM Tris-HCl (pH 8.5) at 60°C for 10min, and subjected to SDS–PAGE with a 16% gel containing 7.5M urea as previously described (Ikeuchi and Inoue). Total protein or isolated PSI-LHCI samples were used for SDS–PAGE. Total proteins were extracted using IP lysis buffer (50 mM Tris-HCl, pH 7.5, 150 mM NaCl, 1mM EDTA, 10% lycerol and 0.1%Trition X-100) with freshly added PMSF (phenylmethysulphonyl fluoride, 2mM) and protease inhibitor cocktail (Roche). Following SDS-PAGE, proteins were transferred to a PVDF membrane (0.45 μm). Membranes were blocked in Tris-buffered solution containing 0.05% Tween 20 (TBS-T) and 5% (w/v) nonfat dry milk for 1 h at room temperature. All antibodies used in this study were purchased from Phyto AB. For primary antibodies of LHCA4 (PhytoAB), TBS-T solutions containing 1% (w/v) nonfat dry milk were prepared to a dilution of 1:10,000 and incubated overnight at 4°C. Subsequently, the membrane was washed three times for 10 min each time in TBS-T. For secondary antibodies, the membrane was incubated for 40 min at room temperature using a goat anti-rabbit IgG (H + L), horseradish peroxidase conjugate (PhytoAB) in TBS-T containing 1% (w/v) nonfat dry milk at a final antibody dilution of 1:10,000. The membrane was then washed three times for 10 min in TBS-T. An ECL luminescence solution was evenly placed over the membrane and incubated for 5 s, and then luminescence was immediately detected with a Tanon-5200 instrument.

### Low-temperature fluorescence spectroscopy

Low-temperature (77K) fluorescence emission spectra were recorded using a F-4700 instrument (HITACHI). The excitation wavelength was 440 nm, and emission was detected in the 600- to 800-nm range. Excitation and emission slit widths were set to 3 nm. Samples were dissolved in 50% (v/v) glycerol, 10 mM Tricine-NaOH (pH 7.5), and 0.015% (v/v) β-DDM. The final concentration of Chls in the sample was 1 μg/mL.

### Absorption spectrometry

Absorption spectra were recorded using a U-3900H spectrophotometer (HITACHI). All PSI-LHCI samples were finally diluted to 4 µg/mL and detected in the 350- to 750-nm range. Samples were dissolved in 10 mM Tricine-NaOH (pH 7.5) and 0.015% β-DDM.

### Measurement of chlorophyll fluorescence induction kinetics curves

Activities of PSII and PSI were measured using a dual-wavelength pulse-amplitude-modulated fluorescence monitoring system (Dual-PAM-100, Walz, Effeltrich, Germany), and all materials were subjected to dark adaptation for 30 min before determination. Parameters were automatically calculated by the Dual-PAM-100 software during the measurement (Klughammer and Schreiber, 2008). First, the minimal fluorescence after dark adaptation (Fo), the maximum fluorescence (Fm) and the maximal change in P700^+^signal (Pm) was determined using a saturation pulse (10,000 μmol photons m^−2^ s^−1^ for 0.3 s). Plants were then continuously illuminated with actinic light (111 μmol photons m^−2^ s^−1^) for 5 min, and saturating pulses were imposed every 20 s. The maximum fluorescence (Fm’), the maximum P700^+^signal (Pm’) in the light-adapted state, and the steady state fluorescence (F) and P700^+^signal (P) during actinic illumination were measured. The actinic light was removed, and the minimal fluorescence level in the light-adapted state (Fo’) was determined by illuminating the leaf with a 3-s pulse of far-red light. The quantum yields of energy conversion in PSI and PSII were calculated with the following equations: Y(I) = (Pm’ − P)/Pm (Li et al., 2021); Y(II) = (Fm’ − F)/Fm’ (Klughammer and Schreiber, 2008; Suzuki et al., 2011).

### Pigment analysis

The pigments were extracted with 80% (v/v) acetone and analyzed by high-performance liquid chromatography (HPLC) (Thermo Fisher, UltiMate 3000). The liquid phase system and the pigment separation method were as previously described (Angeler and Schagerl, 1997).

## Results

### Single amino acid substitutions at the N99 residue of LHCA4 cause a blue shift of the far-red fluorescence emission *in vivo*

We selected four amino acids out of the possible 19 to replace the N99 residue of LHCA4 based on three criteria: 1) stability of the mutant protein, 2) the coordination of the central Mg atom in the mutant protein, and 3) previous *in vitro* results. Accordingly, we calculated the predicted protein stability of LHCA4 harboring a substitution of N99 with one of the other 19 amino acids. We thus selected two mutants each with high and low values: cysteine (C) and methionine (M) for high values, and glycine (G) and histidine (H) for low values (**Table S2**). H is the most common amino acid that provides a ligand to the central Mg atom in Chl (Li et al., 2022); moreover, *in vitro* assays indicated that the N99H mutation still binds to Chl *a*603 (Morosinotto et al., 2003). The other three mutated residues (G, C, and M) might use their main-chain carbonyls to function as ligands to the central Mg atom of Chl *a*603.

**Figure 1:**
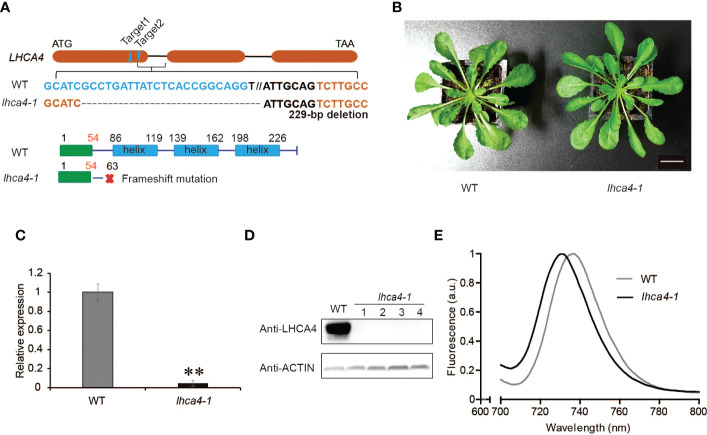
Characterization of the *lhca4-1* mutant. **(A)** Schematic diagram of the *LHCA4* locus showing the target sites of sgRNAs to generate the *lhca4-1* mutant. **(B)** Representative rosette phenotype of the wild type (WT) and *lhca4-1* (scale bars = 2 cm). **(C)** Relative *LHCA4* expression levels as detected by reverse transcription quantitative PCR (RT-qPCR) analysis in the WT and *lhca4-1* (using the two‐tailed Student’s t‐test; **significant at P < 0.01). **(D)** LHCA4 abundance in the WT and four *lhca4-1* plants, as detected by immunoblotting. ACTIN served as loading control. **(E)** Low-temperature (77K) fluorescence emission spectra (excitation at 440 nm) of the WT and *lhca4-1*. The spectra were normalized to their maximal emission, which was set to 1.

To remove any contribution from endogenous LHCA4, we generated an *lhca4-1* mutant by clustered regularly interspaced short palindromic repeat (CRISPR)/CRISPR-associated nuclease 9 (Cas9)-mediated gene editing. To this end, we designed two single guide RNAs (sgRNAs) targeting sequences in *LHCA4*, leading to the isolation of a knockout mutant in the T_2_ generation, *lhca4-1*, harboring a 229-bp deletion in *LHCA4* predicted to introduce a premature stop codon after 63 amino acids (**Figure 1A**). The *lhca4-1* mutant had a very similar appearance as the wild-type (WT) Col-0 under normal growth conditions (**Figure 1B**). Importantly, both *LHCA4* transcript levels and LHCA4 abundance in *lhca4-1* were much lower or undetectable, respectively, indicating that the *lhca4-1* mutant is likely a null allele (**Figures 1C, D**). We determined the fluorescence emission properties of leaves from the WT and *lhca4-1* by low-temperature (77K) fluorescence spectroscopy and observed a clear difference between the two genotypes, with a fluorescence emission maximum of 735 nm in the WT and 730 nm in *lhca4-1*(**Figures 1E**, and **Figure S2A**). The loss of LHCA4 therefore caused a 5-nm blue shift, which is consistent with a previous report (Zhang et al., 1997).

**Figure 2:**
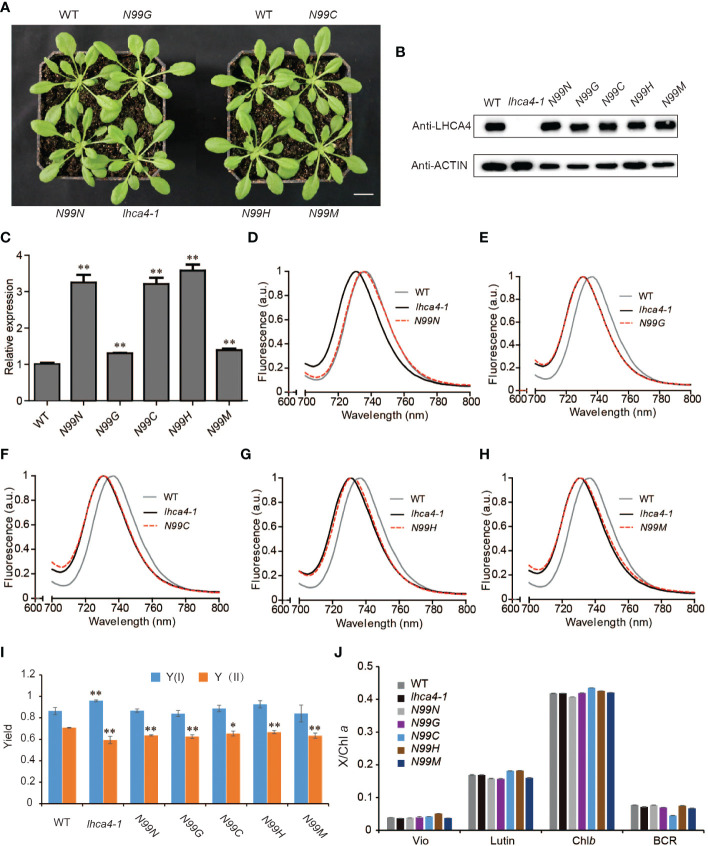
Phenotypic and physiological characterization of plants with single amino acid substitutions at N99 in LHCA4. **(A)** Representative photograph of the WT, *lhca4-1*, the control line *35S:LHCA4*(*N99N*), and four lines harboring LHCA4 point mutations in the *lhca4-1* mutant background: *35S:LHCA4*(*N99G*), *35S:LHCA4*(*N99C*), *35S:LHCA4*(*N99H*), and *35S:LHCA4*(*N99M*) (scale bars = 2 cm). **(B-C)** LHCA4 protein abundance analyzed by immunoblots. Anti-ACTIN antibodies were used for the immunoblots assays. **(B)** and relative *LHCA4* expression levels detected by RT-qPCR (using the two-tailed Student’s t-test; **significant at P < 0.01) **(C)** in 5-week-old plants from the WT, *lhca4-1*, the control line, and the LHCA4 point mutants. **(D-H)** 77K fluorescence emission spectra for the WT, *lhca4-1*, and *35S:LHCA4*(*N99N*) **(D)**; *35S:LHCA4*(*N99G*) **(E)**; *35S:LHCA4*(*N99C*) **(F)**; *35S:LHCA4*(*N99H*) **(G)**; and *35S:LHCA4*(*N99M*) **(H)**. The excitation wavelength was 440 nm. **(I-J)** Chl fluorescence parameters of Y(I) and Y(II) (using the two-tailed Student’s t-test; *significant at P < 0.05, **significant at P < 0.01). **(I)** and pigment composition **(J)** in the WT, *lhca4-1*, the control line, and the four LHCA4 point mutant lines.

To explore the *in vivo* properties of the four selected LHCA4 variant proteins above (N99G, N99C, N99H, and N99M), we placed their respective coding sequences under the control of the strong cauliflower mosaic virus (CaMV) 35S promoter and introduced each construct into the *lhca4-1* mutant. We also introduced the wild-type version of the *LHCA4* coding sequence (N99N) into *lhca4-1* as a control line. We confirmed that all transgenes are present in the *lhca4-1* background (**Figure S3**). We observed no phenotypic differences between the WT and the five types of transgenic lines (**Figure 2A**). An immunoblot assay indicated that LHCA4 abundance in the transgenic lines is comparable to that in the WT (**Figure 2B**), and reverse transcription quantitative PCR (RT-qPCR) showed that the relative *LHCA4* expression leaves are higher in the transgenic lines than in the WT (**Figure 2C**). We also determined the effects of the single amino acid substitutions on fluorescence emission using leaves from each transgenic line and the WT by low-temperature (77K) fluorescence spectroscopy. The control line, harboring the *35S:LHCA4*(*N99N*) transgene, showed a fluorescence emission maximum at 735 nm (**Figure 2D** and **Figure S2A**), which was identical to that in the WT. By contrast, all other transgenic lines carrying the variant constructs 35S:*LHCA4*(*N99G*), *35S:LHCA4*(*N99C*), *35S:LHCA4*(*N99H*), and *35S:LHCA4*(*N99M*) showed similar fluorescence emission spectra peaking at 730 nm, as in the *lhca4-1* mutant (**Figures 2E-H** and **Figure S2A**). We concluded that all substitutions at residue N99 in LHCA4 cause a blue shift in the fluorescence emission of the variant LHCA4 protein, indicating that the excited-state energy level of the red Chls in variant LHCA4s increased when compared to LHCA4 in the WT and the *N99N* control line. While the point mutations almost had no effect on the potential photosynthetic performance, we observed little change in the Chl fluorescence parameters Y(I) and Y(II) in all of the substitutions compared to the control line (**Figure 2I**), indicating that the photochemical quantum yields of PSI and PSII were maintained well based on the fact that the pigment network within PSI was almost unchanged. In addition, high-performance liquid chromatography (HPLC) analysis showed no significant differences among the WT, the control *N99N* line, and the other transgenic lines in their pigment/Chl *a* ratio (**Figure 2J**). These results suggest that mutating N99 in LHCA4 to G, C, H, or M has little effect on plant phenotypes and photochemical vitality, although we detected a distinct effect on the energy level of red Chls in LHCA4.

### The PSI-LHCI complex of *LHCA4* transgenic lines includes LHCA4

**Figure 3:**
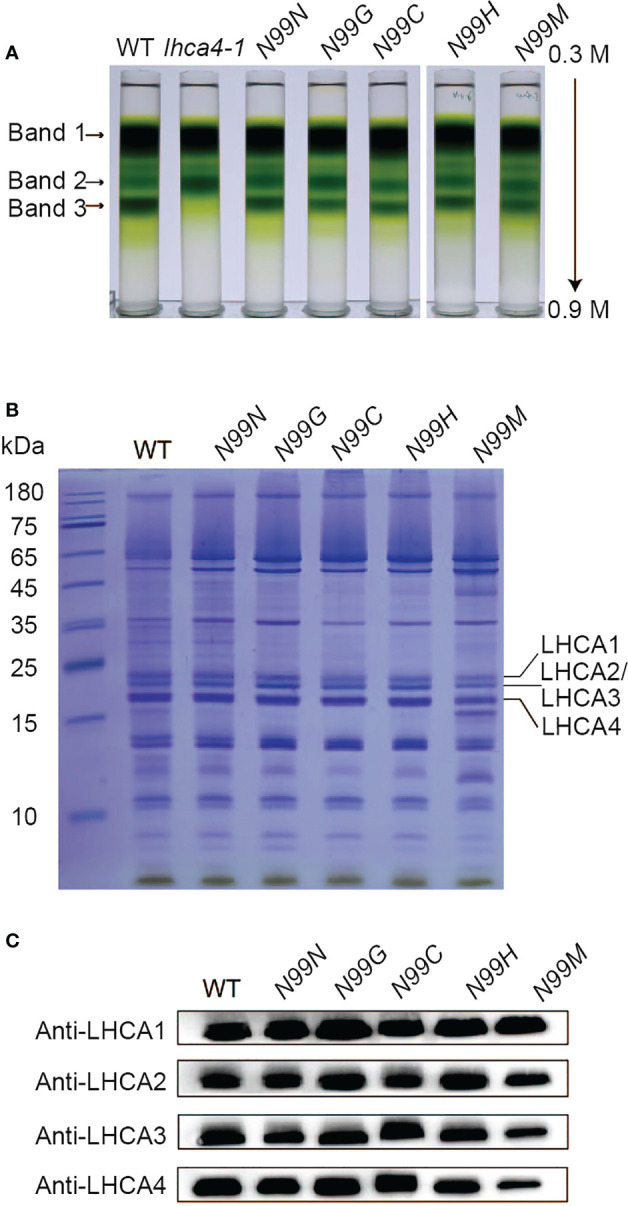
Isolation and characterization of PSI-LHCI from N99 point mutant lines. **(A)** Sucrose density gradient ultracentrifugation of detergent-treated thylakoid membranes isolated from the WT, *lhca4-1*, the control, and N99 point mutant lines. The lowest band, labeled Band 3, was collected and analyzed. **(B-C)** Polypeptide composition of Band 3 as analyzed by Coomassie staining after SDS-PAGE (samples corresponding to 2.5 μg Chl were loaded per lane). **(B)** and immunoblot analysis with antibodies against LHCA1, LHCA2, LHCA3, and LHCA4 **(C)**.

To assess whether the variant LHCA4 proteins were assembled into the PSI-LHCI complex in the transgenic lines, we isolated PSI samples through sucrose density gradient ultracentrifugation of detergent-treated thylakoid membranes (**Figure 3A**). The WT thylakoid membrane separated into three major bands, corresponding to dissociated antenna proteins (Band 1), PSI core and PSII core (Band 2), and PSI-LHCI (Band 3) based on SDS-PAGE of each fraction (**Figure 3** and **Figure S4**). Band 3 was much wider and darker than Band 2, suggesting that most of the PSI core is in a large super-complex associated with antenna proteins. Notably, the *lhca4-1* mutant had no visible Band 3, with Band 1 migrating at the same position in the gradient as in the WT, while Band 2 was much darker than in the WT. SDS-PAGE and immunoblot analyses determined that LHCA2 and LHCA3, but not LHCA1 or LHCA4, comprise Band 2 (**Figure S4**). For all transgenic lines expressing LHCA4 variants at N99, we detected all three bands at the same position as in the WT. However, the Chl contents of Bands 2 and 3 differed between the transgenic lines and the WT, with Band 2 having 21–60% more Chls than the WT and Band 3 accumulating about 20% less Chls than the WT, yielding a darker Band 2 and lighter Band 3 in the transgenic lines (**Table S3**). Band 2 from the mutants contained PSI core proteins and part of LHCA proteins (**Figure S4**), while Band 3 contained all four LHCA proteins (LHCA1 to LHCA4), as determined by SDS-PAGE and immunoblot analysis (**Figures 3B, C**). Therefore, although most of the PSI core assembled into a full-size PSI-LHCI complex, we speculate that a small fraction of PSI failed to fully assemble with LHCI due to the absence of LHCA4 and migrated into Band 2, thus explaining its higher Chl contents. To assess whether the lower intensity of Band 3 in the transgenic lines expressing the *LHCA4* variants was a consequence of the mutations, we tested the *N99N* control line under the same conditions and observed a pattern similar to that of the transgenic lines (**Figure 3A**, **Table S3**), suggesting that the decrease in fully assembled PSI-LHCI complex (Band 3) is not a consequence of the point mutations but may instead be caused by the introduction of the transgene. We thus propose that the single amino acid substitutions at the N99 residue in LHCA4 did not affect its binding to the PSI core.

### Single amino acid substitutions at the N99 residue in LHCA4 cause a blue shift of the far-red fluorescence emission of PSI-LHCI

**Figure 4:**
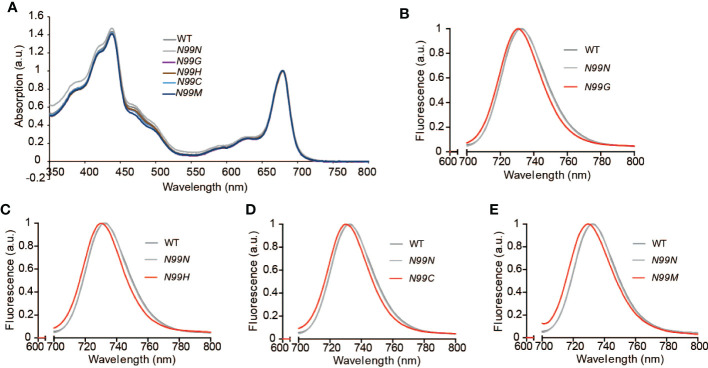
Spectroscopic properties of PSI-LHCI isolated from the WT and N99 point mutant lines. **(A)** Absorption spectra at room temperature of the WT and N99 point mutant lines. The spectra were normalized, with their maximal absorption in the Qy region set to 1. **(B-E)** Low-temperature (77K) fluorescence emission spectra of PSI-LHCI complexes (Band 3) isolated from the WT (dark grey solid line), the control line *35S:LHCA4*(*N99N*) (light gray solid line), and the N99 point mutant lines *35S:LHCA4*(*N99G*) **(B)**, *35S:LHCA4*(*N99H*) **(C)**, *35S:LHCA4*(*N99C*) **(D)**, and *35S:LHCA4*(*N99M*) **(E)**, shown as red solid lines. The excitation wavelength was 440 nm.

To explore how the amino acid substitutions introduced in LHCA4 at the N99 residue affected the spectroscopic properties of PSI-LHCI, we measured the absorption spectra and the low-temperature fluorescence emission spectra of PSI-LHCI isolated from WT and N99 mutant plants. PSI-LHCI from the WT and all *LHCA4* transgenic lines showed similar room temperature absorption spectra (**Figure 4A**), although they differed in their low-temperature fluorescence emission spectra (**Figures 4B-E**, **Figure S2B**). Indeed, PSI-LHCI isolated from the WT had a fluorescence emission peak at 732 nm, exhibiting a 3-nm blue shift compared to its 77K fluorescence profile when embedded in thylakoid membranes. PSI-LHCI isolated from the control line *N99N* showed a fluorescence emission peak at 732.2 nm, which was similar to that seen for PSI-LHCI in WT plants (**Figure 4B**). By contrast, all four transgenic lines with point mutations in N99 showed fluorescence emission peaks at 730.6 nm (*N99G*, **Figure 4B**), 730 nm (*N99C*, **Figure 4C**), 730.4 nm (*N99H*, **Figure 4D**), and 729.8 nm (*N99M*, **Figure 4E**), with an ~2-nm blue shift compared to PSI-LHCI from WT plants and the *N99N* control line. This result suggested that replacing N99 with G, C, H, or M in LHCA4 causes a moderate blue shift that is difficult to detect at room temperature by absorption spectroscopy due to the low ratio of red Chls. The observed blue shift in the red Chl forms may reflect a change in the geometrical arrangement of Chl *a*603, which would lead to a concomitant change in the interaction between Chl *a*603 and Chl *a*609. Another possibility is that no Chl molecule bound to the Chl *a*603 site due to the mutated residue at position 99, leaving only Chl *a*609 to bind, which would abolish the far-red absorption of the PSI-LHCI complex. We conclude that the native N99 residue of intact LHCA4 gives rise to the strongest far-red absorption and greatest red shift, while other residues at this position decrease the far-red absorption of the complex.

### Changing E146 to Q in LHCA4 broadens the far-red emission window into the red

**Figure 5:**
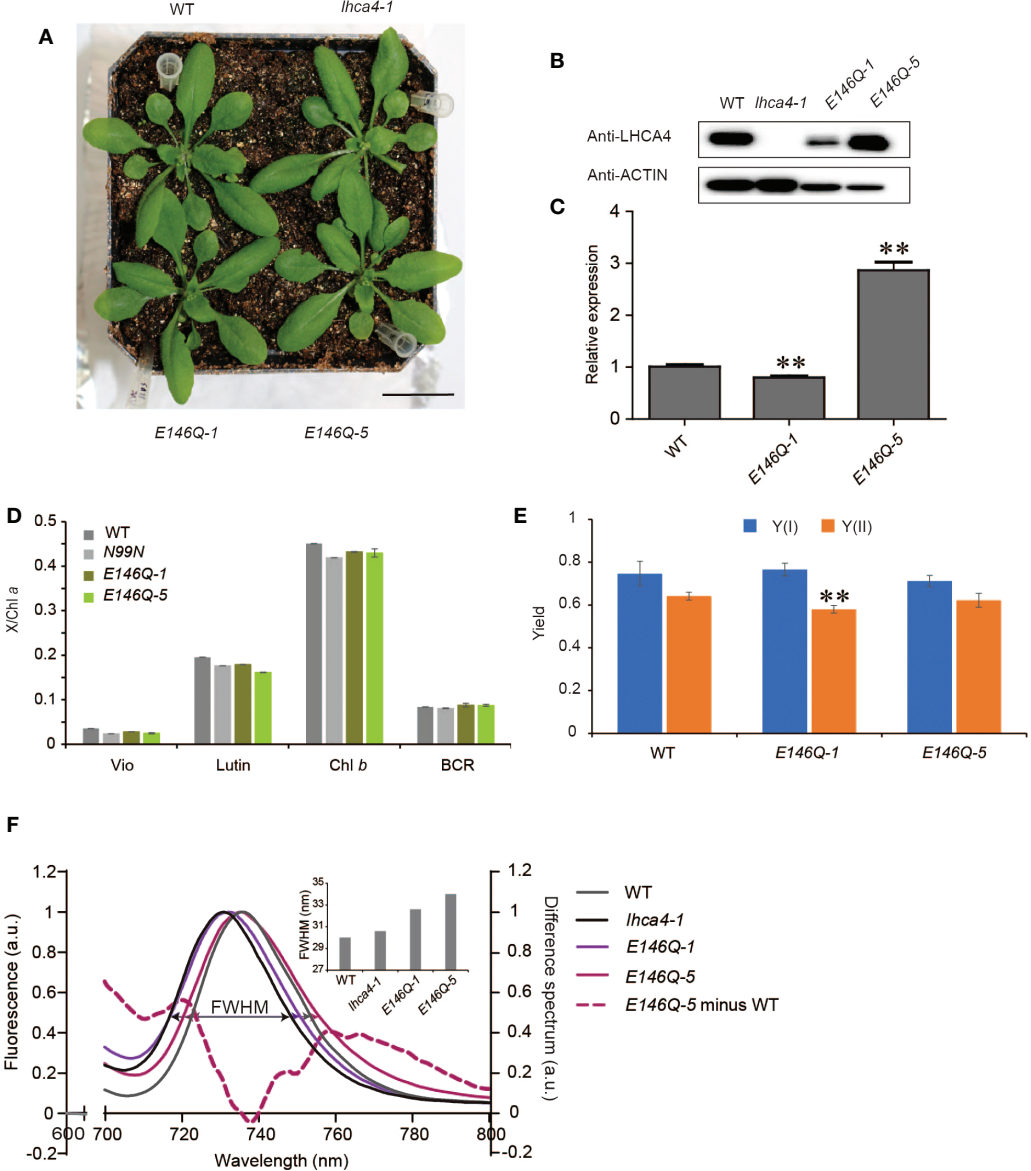
Characterization of LHCA4(E146Q) point mutant lines. **(A)** Phenotype of the WT, *lhca4-1*, and two LHCA4(E146Q) point mutant lines, *35S:LHCA4*(*E146Q*)*-1* and *35S:LHCA4*(*E146Q*)*-5* (scale bars = 2 cm). **(B-C)** LHCA4 abundance as detected by immunoblot analysis. Anti-ACTIN antibodies were used for the immunoblots assays. **(B)** and relative *LHCA4* expression levels as detected by RT-qPCR (using the two-tailed Student’s t-test; **significant at P < 0.05) **(C)** in the WT, *lhca4-1*, and LHCA4(E146Q) point mutant lines. **(D-E)** Pigment composition **(D)** and chlorophyll fluorescence parameters Y(II) and Y(I) **(E)** of the WT and LHCA4(E146Q) point mutant lines (using the two-tailed Student’s t-test; **significant at P < 0.01). **(F)** Low-temperature (77K) fluorescence emission spectra (excited at 440 nm) of the WT (gray), *lhca4-1* (black), *35S:LHCA4*(*E146Q*)*-1* (light purple), and *35S:LHCA4*(*E146Q*)*-5* (dark purple). The purple dotted line shows the difference between the spectra of *35S:LHCA4*(*E146Q*)*-5* and the WT (and multiplied by 5 to facilitate comparison). The FWHMs (full widths at half maximum) for each line are labeled and marked with double arrows in the same colors as the spectra.

The *in vitro* assays determined that the E146Q substitution in LHCA4 resulted in a strongly red-shifted emission by 10 nm, from 731 to 741 nm relative to the WT (Wientjes et al., 2012), indicating that E146 might be a candidate to broaden the absorption properties of PSI-LHCI complexes toward the red region of the spectrum *in vivo*. To determine how the replacement of E146 by Q in LHCA4 would affect the far-red absorption *in vivo*, we introduced the *35S:LHCA4*(*E146Q*) construct into the *lhca4-1* mutant and obtained two transgenic lines with a WT appearance: *35S:LHCA4*(*E146Q*)*-1* and *35S:LHCA4*(*E146Q*)*-5* (**Figure 5A** and **Figure S3**). Immunoblot and RT-qPCR analysis indicated that *35S:LHCA4*(*E146Q*)*-1* has a lower LHCA4(E146Q) abundance than LHCA4 in the WT, while *35S:LHCA4*(*E146Q*)*-5* accumulated more LHCA4(E146Q) than LHCA4 in the WT (**Figures 5B, C**). However, their pigment composition and potential photosynthetic performance were similar to those of the WT (**Figures 5D, E**). Moreover, the leaves of *E146Q* transgenic lines showed different low-temperature fluorescence spectra, as evidenced by their respective emission maxima and the full width at half maximum (FWHM) values. Indeed, the emission maximum of leaves from line *E146Q-5* was 735.2 nm, which was very close to that of the WT (735.6 nm), while the emission maximum of leaves from line *E146Q-1* was 732 nm, 2 nm red-shifted than that of the *lhca4-1* mutant (**Figure 5F**). In addition, the FWHM values in lines *E146Q-1* and *E146Q-5* were 32.6 and 34 nm, respectively, both of which covering a larger window than the WT (30 nm) or the *lhca4-1* mutant (30.6 nm) (**Figure 5F**). To more clearly illustrate the difference between line *E146Q-5* and the WT, we calculated the difference spectrum between the two genotypes and multiplied the results by five to raise the amplitude. We observed two positive peaks, one from 710 to 735 nm with a maximum at 721 nm, and the other from 745 to 800 nm with a maximum at 766 nm (**Figure 5F**). Therefore, although the emission maximum of line *E146Q-5* was similar to that of the WT, its emission peak was broader by increasing the emission at the two above regions when compared to the WT. The region with the peak at 766 nm indicated that line *E146Q-5* has greater absorption in a more red-shifted region than the WT, which may be attributed to a much lower energy level of red Chls in LHCA4 (E146Q) than in intact LHCA4. By contrast, substitutions at the N99 residue in LHCA4 had less effect on the FWHM values no matter the fluorescence emission spectra were detected with leaves or isolated PSI-LHCI samples (**Table S4**). In summary, the E146Q substitution in LHCA4 enhanced and broadened the far-red absorption window of plants, suggesting that changing the micro-environment around one of the red Chls, Chl *a*609, might increase far-red absorption.

**Figure 6:**
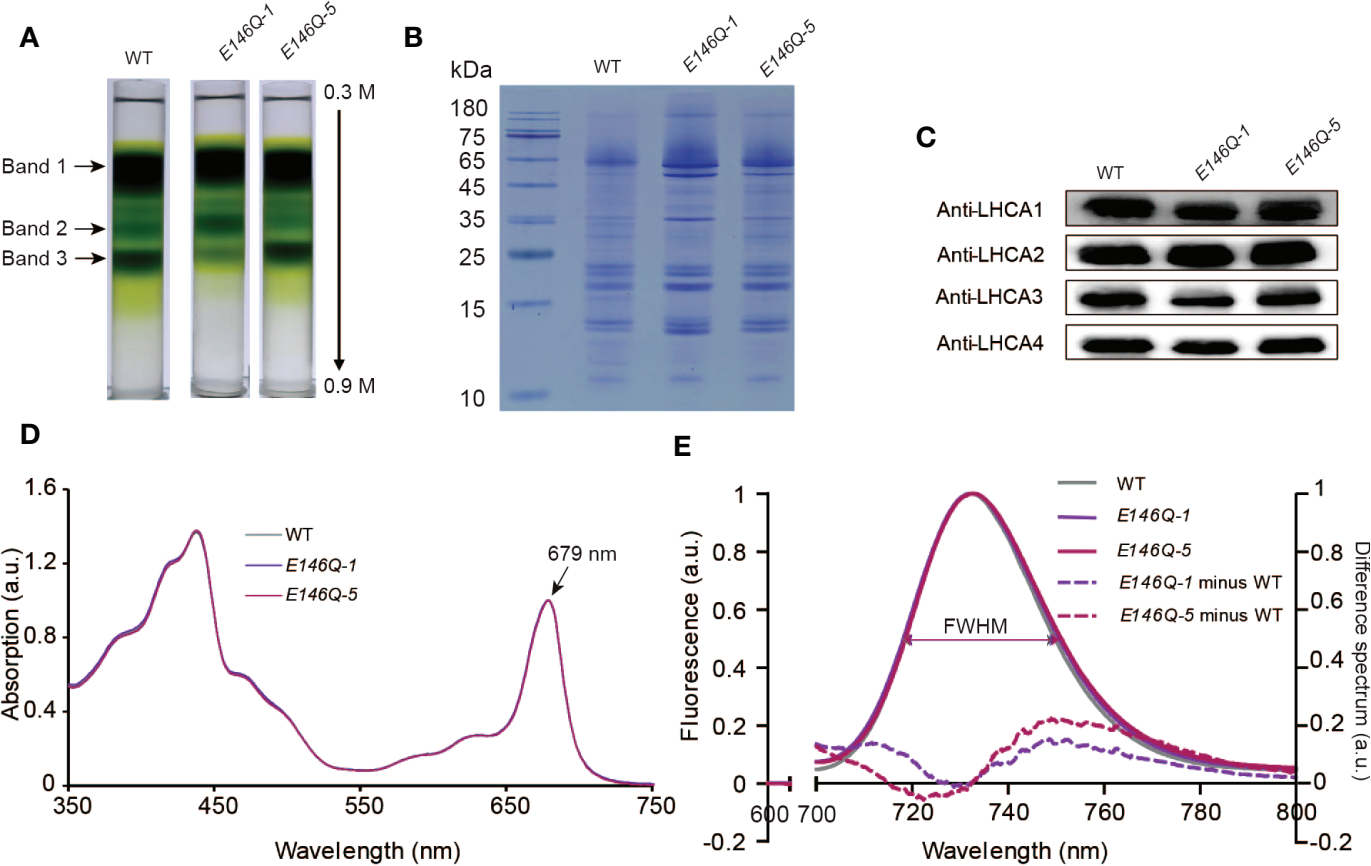
Isolation and characterization of PSI-LHCI from *35S:LHCA4*(*E146Q*) point mutant plants. **(A)** Sucrose density gradient (0.3–0.9 M) ultracentrifugation of thylakoid membranes isolated from WT and *35S:LHCA4*(*E146Q*) point mutant lines. Samples corresponding to 0.5 mg Chls were loaded onto each tube, and three major bands were separated. **(B-C)** Polypeptide composition of PSI-LHCI from the WT and *35S:LHCA4*(*E146Q*) lines analyzed by Coomassie staining following SDS-PAGE (samples corresponding to 2.5μg Chl were loaded per lane). **(B)** and immunoblot analysis **(C)** with antibodies against LHCA1, LHCA2, LHCA3, and LHCA4. **(D)** Absorption spectra of PSI-LHCI complexes isolated from the WT and *35S:LHCA4*(*E146Q*) lines. The spectra were normalized to their maximal absorption in the Qy region, which was set to 1. **(E)** Low-temperature (77K) fluorescence emission spectra (excited at 440 nm) of PSI-LHCI complexes isolated from the WT and *35S:LHCA4*(*E146Q*) lines. The spectra were normalized to their emission maxima (set to 1), with their FWHMs labeled and shown as double arrows in the same colors as the spectra. The dotted lines show the difference between the spectra from *35S:LHCA4*(*E146Q*)*-1* and the WT (light purple line) or *35S:LHCA4*(*E146Q*)*-5* and the WT (dark line) and multiplied by 5 to facilitate comparison.

To determine the effect of the E146Q substitution in LHCA4 on the far-red absorption at the level of the PSI-LHCI complex, we isolated PSI-LHCI complexes from lines *E146Q-1* and *E146Q-5* by sucrose density gradient ultracentrifugation. We observed the same three major bands in these transgenic lines as in the WT (**Figure 6A**). Band 3 in the two transgenic lines displayed the same full-size PSI-LHCI complex as the WT (**Figures 6B, C**), although the associated Chl differed, with Band 3 in line *E146Q-1* having less Chls than the WT or line *E146Q-5* (**Figure 6A**), which was consistent with the lower accumulation of LHCA4 in line *E146Q-1* (**Figure 5B**). These results suggest that LHCA4 with the E146Q substitution can bind to the PSI core and form a PSI-LHCI complex, with a yield of PSI-LHCI complex formation that depends on LHCA4 abundance.

We established that isolated PSI-LHCI complexes from the WT and lines *E146Q-1* and *E146Q-5* have similar absorption spectra, all with an absorption maximum at 679 nm in the red region (**Figure 6D**), indicating that the E146Q substitution did not affect the pigment composition of the PSI-LHCI complex. In terms of fluorescence emission spectra, the PSI-LHCI complexes from lines *E146Q-1* and *E146Q-5* showed peaks at 732.6 and 733.2 nm, respectively, which were close to those in the WT. We noticed an important difference in the FWHMs of the three genotypes: the FWHM from the WT was 31.0 nm, while the FWHMs from *E146Q-1* and *E146Q-5* were 32.4 and 32.2 nm, respectively, indicating that isolated PSI-LHCI complexes with the E146Q substitution have a 1- to 2-nm wider FWHM than the intact PSI-LHCI complex. To better illustrate this difference, we calculated the difference spectrum between *E146Q-1* and the WT and between *E146Q-5* and the WT as above. We observed a positive peak in the 733- to 800-nm range with a maximum at 748.8 nm, indicating that PSI-LHCI with the E146Q substitution in LHCA4 displays an increased fluorescence emission at longer wavelengths (**Figure 6E**). As the increased fluorescence emission came from the red absorption form only, we concluded that the E146Q substitution in LHCA4 enhances the far-red absorption of the PSI-LHCI complex, in agreement with the results obtained with leaves. Notably, the extent of FWHM broadening varied between plants and their isolated PSI-LHCI complexes, with a smaller broadening observed when PSI-LHCI was dissociated from the thylakoid membrane (**Figures 5F** and **6E**). We conclude that the E146Q substitution in LHCA4 increases fluorescence emission into the red part of the spectrum, which was easier to detect in leaves than in isolated PSI-LHCI complexes, suggesting that changes in the micro-environment around red Chl may affect its far-red absorption properties.

## Discussion

Because the red forms of LHCAs originated from the excitonically coupled Chl *a*603-*a*609 dimer mixed with a charge-transfer state (Romero et al., 2009), changes in the organization of the Chl dimer or in the protein environment might influence the energy level of red forms. In this work, we investigated the influence of single amino acid substitutions at amino acid positions 99 and 146 in LHCA4 on the energy levels of the red forms *in vivo* to clarify how the red forms are regulated.

The transgenic lines with the single substitutions N99G, N99C, N99H, or N99M in LHCA4 exhibited similar fluorescence emission spectra as the *lhca4-1* mutant generated by genome editing (**Figure 1E**), as well as the *LHCA4* antisense mutant previously reported (Zhang et al., 1997). Moreover, all transgenic lines (outside of the N99N control line) showed a blue shift of 5–6 nm when compared to the WT (**Figures 2D-H**). To ascertain that the blue-shifted emission of these transgenic lines harboring single substitutions at N99 was solely the reflection of an emission blue shift of the PSI-LHCI complex, we examined the extent of the shift from PSI-LHCI complexes isolated from the transgenic lines. We confirmed that the substitutions did not prevent the formation of a full-size PSI-LHCI complex (**Figure 3**), supporting the notion that any observed shift in emission can be attributed to the introduced mutations.

The fluorescence emission maximum of WT leaves was not identical to that of its corresponding purified PSI-LHCI complex. Indeed, the PSI-LHCI complex isolated from WT leaves had a fluorescence emission peak at 732.4 nm, which was blue-shifted by 3 nm relative to the peak measured in WT leaves (**Figure 2D**). This observation suggested that the embedding of PSI-LHCI particles into the thylakoid membrane may help the complex remain in its most red-shifted state, further raising the possibility that the red forms are sensitive to conformational or a surrounding micro-environmental change caused by dissociation from the thylakoid membrane. By contrast, the fluorescence emission peaks of PSI-LHCI complexes purified from the N99 substitution lines were around 730 nm (**Figures 4B-E**), which was almost identical to those from leaves for their respective plant materials (**Figures 2E-H**). All N99 substitutions in LHCA4 induced a 5-nm blue shift from 735 to 730 nm in leaves (**Figures 2D-H**) and a 2- to 3-nm blue shift from 732.4 to 730 nm in isolated PSI-LHCI complexes, relative to the WT (**Figures 4B-E**). Importantly, the transgenic line harboring the *N99N* construct showed no difference with the WT, indicating that the N99 residue is essential for maintaining the red form of the complex. This result is in agreement with the *in vitro* findings that substitutions of the N99 residue led to the loss of the red form (Morosinotto et al., 2003; Wientjes et al., 2012). These results suggest that none of the substitutions of the Chl *a*603 ligand in LHCA4 can maintain a proper geometry between Chl *a*603 and Chl *a*609 to allow for their strong interaction, thus leading to the observed red shift seen for the PSI-LHCI complex in its free from *in vitro* or embedded in the thylakoid membrane. The surrounding micro-environment of the Chl *a*603-*a*609 pair may affect the red forms. Indeed, fluctuations between conformations with and without red forms in LHCA were detected by single-molecule spectroscopy in a natural state of the isolated LHCA1-LHCA4 dimer (Kruger et al., 2011). The PSI-LHCI structure of pea revealed that many lipids can bind at the gap region between LHCA4 and the PSI core and that the interaction between LHCA4 and the core is the most vulnerable to the alkaline pH among interactions between LHCI and the core (Wang et al., 2021). Interestingly, amino acid 99 of LHCA4 is located at the interface between LHCA4 and the core and may therefore affect the interaction between LHCA4 and the core in response to changes in the local micro-environment.

The TMH region of LHCA4 contains five Glu residues, of which three form ion pairs with Arg residues to stabilize the protein structure, one binds on the lumenal side of helix B, and one in helix C (E146) is close to Chl *a*609 and interacts with Chl *b*607. We investigated the effect of the E146Q substitution on the absorption and emission properties of the red forms *in vivo* by isolating two transgenic lines expressing *LHCA4*(*E146Q*), lines *E146Q-1* and *E146Q-5*, with lower or higher relative *LHCA4* expression levels than the WT, respectively (**Figures 5B, C**). Although the emission peak of line *E146Q-5* was almost identical to that of the WT, around 735 nm, the transgenic lines showed a broader emission into the red part of the spectrum (**Figure 5F**). By contrast, line *E146Q-1* exhibited a blue-shifted emission similar to that of the *lhca4-1* mutant (**Figure 5F**). We propose that the blue shift in line *E146Q-1* was caused by the low LHCA4 abundance rather than by the substitution, as isolated PSI-LHCI complexes from both *E146Q* lines showed no blue shift in their emission spectra when compared to intact PSI-LHCI from the WT, with the mutant-isolated complexes even showing a slight red shift compared to the WT complexes (**Figure 6E**). Indeed, leaves from line *E146Q-5* had a broader absorption window in the red part of the spectrum than its corresponding isolated PSI-LHCI complexes, suggesting that the embedding PSI-LHCI complexes incorporating the LHCA4(E146Q) substitution into the thylakoid membrane contributed to maintaining the red form.

However, compared to the large red shift seen in rLHCA4 with E146Q (Wientjes et al., 2012), the red shift of PSI-LHCI isolated from leaves with the E146Q substitution in LHCA4 was not significant, likely reflecting the differential protein environment surrounding the red dimer of Chls *a*603–*a*609. We propose two hypotheses to explain the large red shift seen in rLHCA4 with the E146Q substitution: 1) the change in charge distribution caused by replacing negatively charged E with neutral Q and 2) one more Chl *b* binding into the protein, thus favoring a more red rLHCA4 conformation. In the context of the PSI-LHCI complex, LHCA4 with the E146Q substitution was not free but interacted with the PSI core and adjacent LHCAs, perhaps making it more difficult to insert additional Chl molecules due to steric hindrance. Importantly, both *in vitro* and *in vivo* experiments agreed that the E146Q substitution showed the same red shift in emission properties, although to a different extent. We thus speculate that the replacement of the negatively charged E in the surrounding environment of Chl *a*609 can change the local environment and enhance the red forms, offering a means to broadening absorption into the far-red region of the spectrum.

Improving energy utilization efficiency has been an important goal of photosynthetic research. Plants are much less effective at absorbing and utilizing far-red light than visible light, which can be a problem in a far-red light–enriched environment such as within or under canopies. Improving far-red light utilization could be an approach to increasing crop production under suboptimal conditions. Oxygenic photosynthetic organisms have two main strategies to use far-red light. In some cyanobacteria, Chl *d* and Chl *f* are produced to capture far-red light; most algae and land plants do not synthesize other Chls outside of Chl *a* and Chl *b*, so they capture far-red light by shifting the absorption spectrum of Chl *a* to a more red region (Wolf and Blankenship, 2019). While engineering a crop that can better utilize far-red light may borrow either of the two above strategies, the introduction of Chl *d* and Chl *f* might prove more difficult, as it would require the biosynthesis of new pigments. Conversely, while the second approach would be easier to implement, a serious limitation is the modest extent of red-shifted absorption compared to cyanobacterial Chl *d* and Chl *f*. The aim of this study was to determine how much the red forms can be shifted *in vivo* when changing amino acids surrounding the red Chls.

In this research, all N99 substitutions tested exhibited a blue shift in their fluorescence emission, suggesting that it might be difficult to engineer a more red-shifted absorbing form by replacing the N99 residue of LHCA4. However, targeting E146 in LHCA4 may be a promising method to expanding the absorption wavelength of LHCA4 and PSI-LHCA complexes into the far-red region. Evolutionarily, the amino acid coordinating with Chl *a*603 has changed from His in green algae such as *Chlamydomonas reinhardtii* (Su et al., 2019) and *Bryopsis corticulans* (Qin et al., 2019), and in the moss *Physcomitrium patens* (Yan et al., 2021), to Asn in angiosperms, concomitantly with a red shift in fluorescence emission maxima from 710 nm in green algae to 727 nm in the moss, and to 735 nm in angiosperms. This evolutionary pattern suggests that land plants have selected LHCA sequences to achieve a lower energy level for red Chls. It may therefore be easier to engineer crop plants with a blue shift in their absorption maxima relative to making red-shifted crops, as land plants appear to have evolved over billions of years to lower the energy level of their PSI-LHCs. In angiosperms, the function of the uphill energy transfer from low-energy Chls to bulk Chls with higher energy is still not well understood, but may be related to adaptation to the complex light environment on land. We speculate that engineering blue-shifted plants might provide a means to improving crop production under artificial light environments, as a weakened uphill energy transfer may lead to a faster EET. As the first exploration of the effects of amino acids surrounding Chl *a*603 and Chl *a*609 on their energy levels *in vivo*, this study provides new information on the relationship between structure and function of red Chls. Continued research on this topic is needed to clarify the mechanisms that tune the energy level of red forms and to design red-shifted or blue-shifted crops to make full use of limited light.

## Data availability statement

The datasets presented in this study can be found in online repositories. The names of the repository/repositories and accession number(s) can be found in the article/**Supplementary Material**.

## Author contributions

XQ planned and designed the research, analyzed the data and wrote the paper. XL and LZ performed the experiments, analyzed the data and wrote the manuscript. JS, GY and CH assisted in performing the experiments. TK and WW analyzed the data. All authors contributed to the article and approved the submitted version.
