# Supplementary material for: LHCA4 residues surrounding red chlorophylls allow for fine-tuning of the spectral region for photosynthesis in Arabidopsis thaliana
Source: Front Plant Sci. 2023 Jan 17;13:1118189. doi: 10.3389/fpls.2022.1118189 (PMC9887303; doi:10.3389/fpls.2022.1118189)
Supplement: Supplementary file 1 [file DataSheet_1.docx]

Supplementary Material

LHCA4 residues surrounding red chlorophylls allow for fine-tuning of the spectral region for photosynthesis in *Arabidopsis thaliana*

Xiuxiu Li^1,2^, Lixia Zhu^2^, Jince Song^2^, Wenda Wang^3^, Tingyun Kuang^3^, Gongxian Yang^2^, Chenyang Hao^2^, Xiaochun Qin^2*^

^1^ School of Chemistry and Chemical Engineering, University of Jinan, Jinan 250022, China.

^2^ School of Biological Science and Technology, University of Jinan, Jinan 250022, China

^3^ Photosynthesis Research Center, Key Laboratory of Photobiology, Institute of Botany, Chinese Academy of Sciences, Beijing 100093, China

*** Correspondence:** Corresponding Author: [bio_qinxc@ujn.edu.cn](mailto:bio_qinxc@ujn.edu.cn)

**Figure S1 Location of amino acids targeted by point mutations and the structure near red Chls in LHCA4.**

**Figure S2 Low temperature (77K) fluorescence emission spectra covering the 600- to 800-nm range.**

**Figure S3 Principles of primer design for mutant detection and detection of point mutant plants in the T1 generation.**

**Figure S4 Polypeptide composition of Band 2 from WT, lhca4-1, and N99 point mutant lines.**

**Table S1 Primers used in this study.**

**Table S2 Prediction of protein stability.**

**Table S3 Photodensity of sucrose density gradient ultracentrifugation.**

**Table S4 FWHMs of 77K fluorescence emission spectra of plants with single amino acid substitutions at N99 in LHCA4 and PSI-LHCI isolated from these plants.**


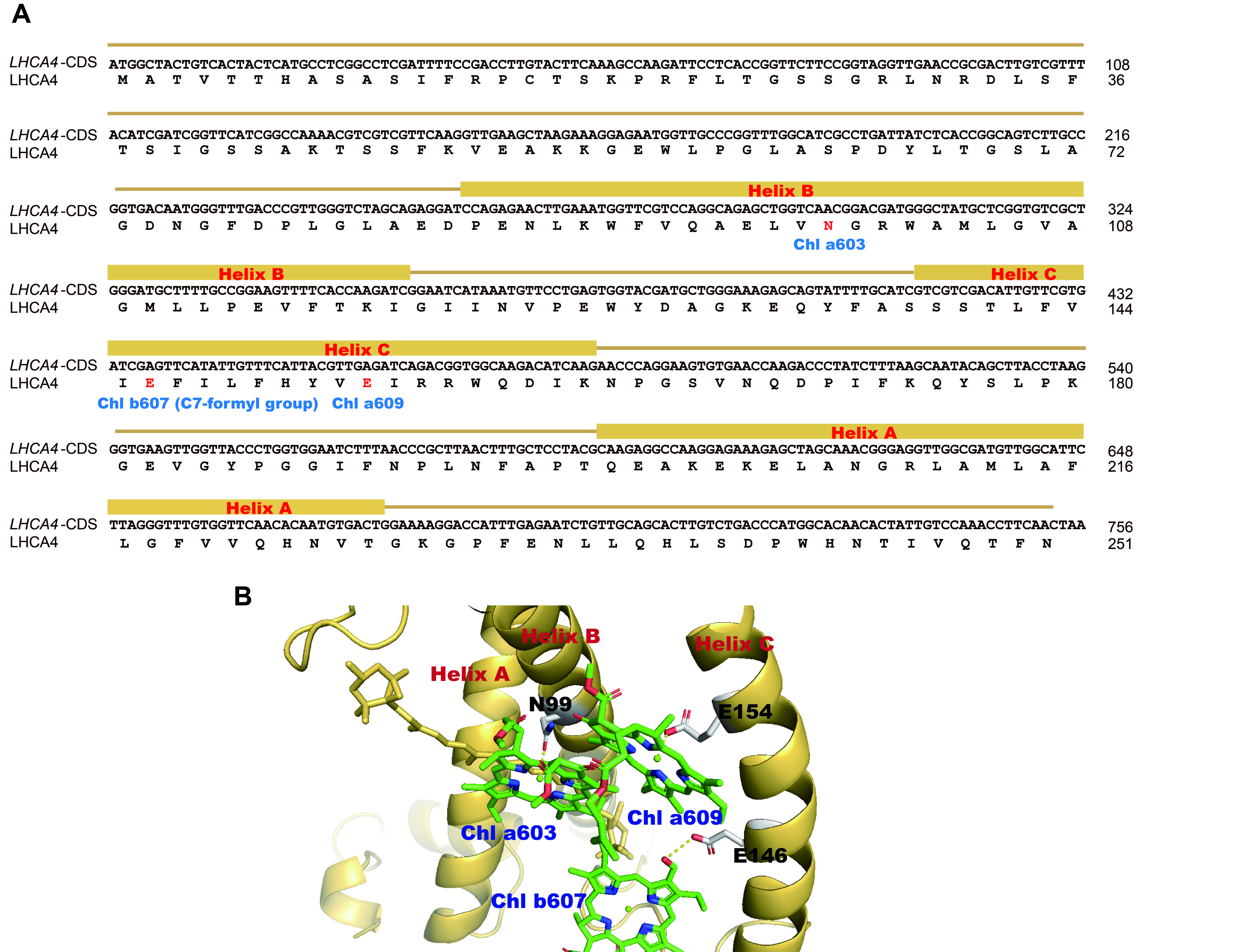


**Figure S1** **Location of amino acids targeted by point mutations and the structure near red Chls in LHCA4.** **(A)** DNA sequence of *LHCA4* and corresponding protein sequence of LHCA4. The brown boxes indicate the three transmembrane helices (Helix A, B, and C). Two amino acids coordinated to Chl *a*603 (N99) and Chl *a*609 (E154) and amino acid E146 forming a hydrogen bond with the C7 formyl group of Chl *b*607 are labeled in red. **(B)** Structure of LHCA4 bound to Chls (Chl a603, Chl a609 and Chl b607) (according to (Qin et al., 2015); PDB ID: 4XK8). Amino acids interacting with Chls are marked in black, and the names of helices are marked in red.


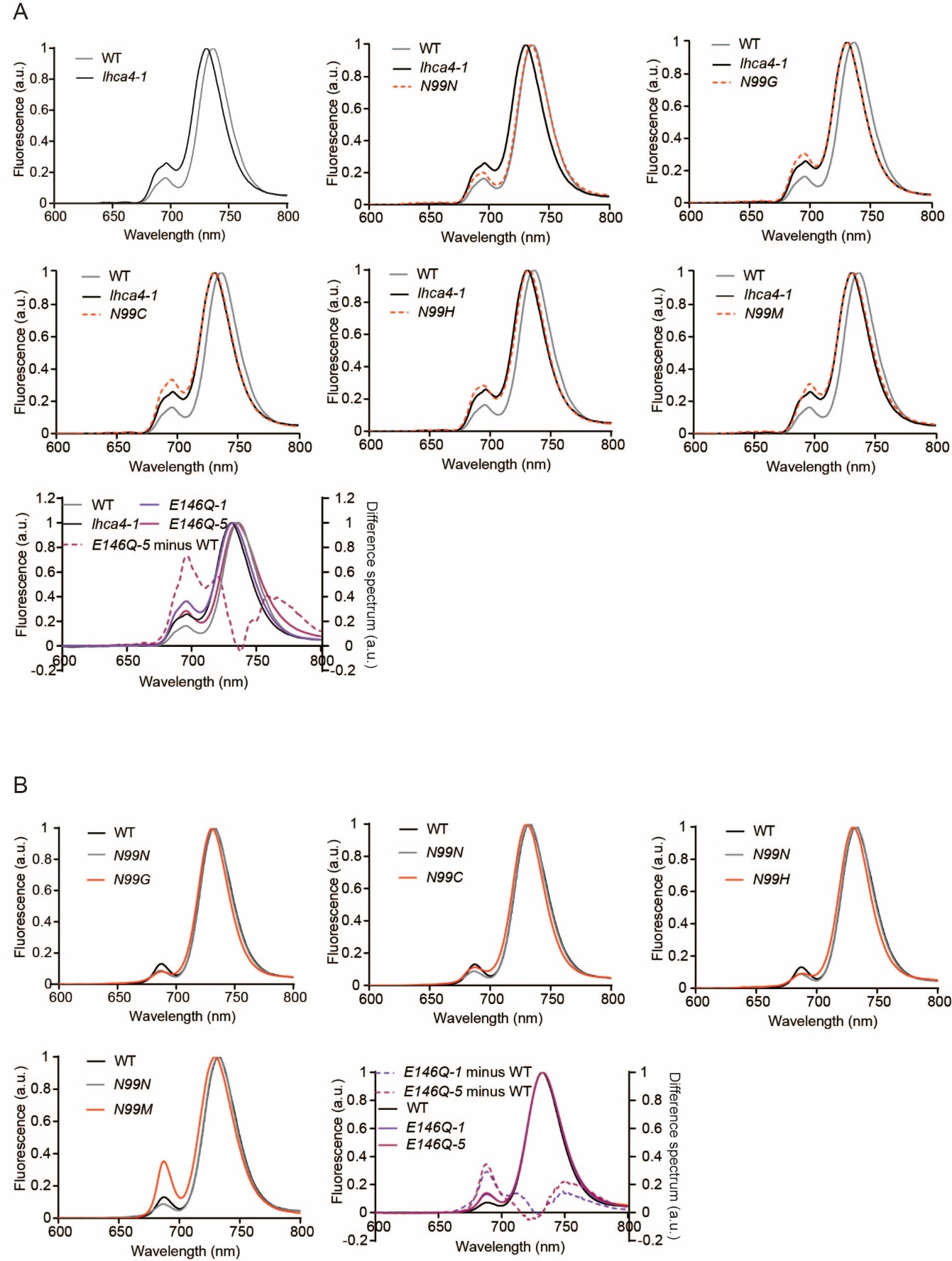


**Figure S2 Low temperature (77K) fluorescence emission spectra covering the 600- to 800-nm range.** (A) Fluorescence emission spectra of leaves with single amino acid substitutions at N99 and E146 in LHCA4. (B) Fluorescence emission spectra of PSI-LHCI isolated from these plants. The excitation wavelength was 440 nm.


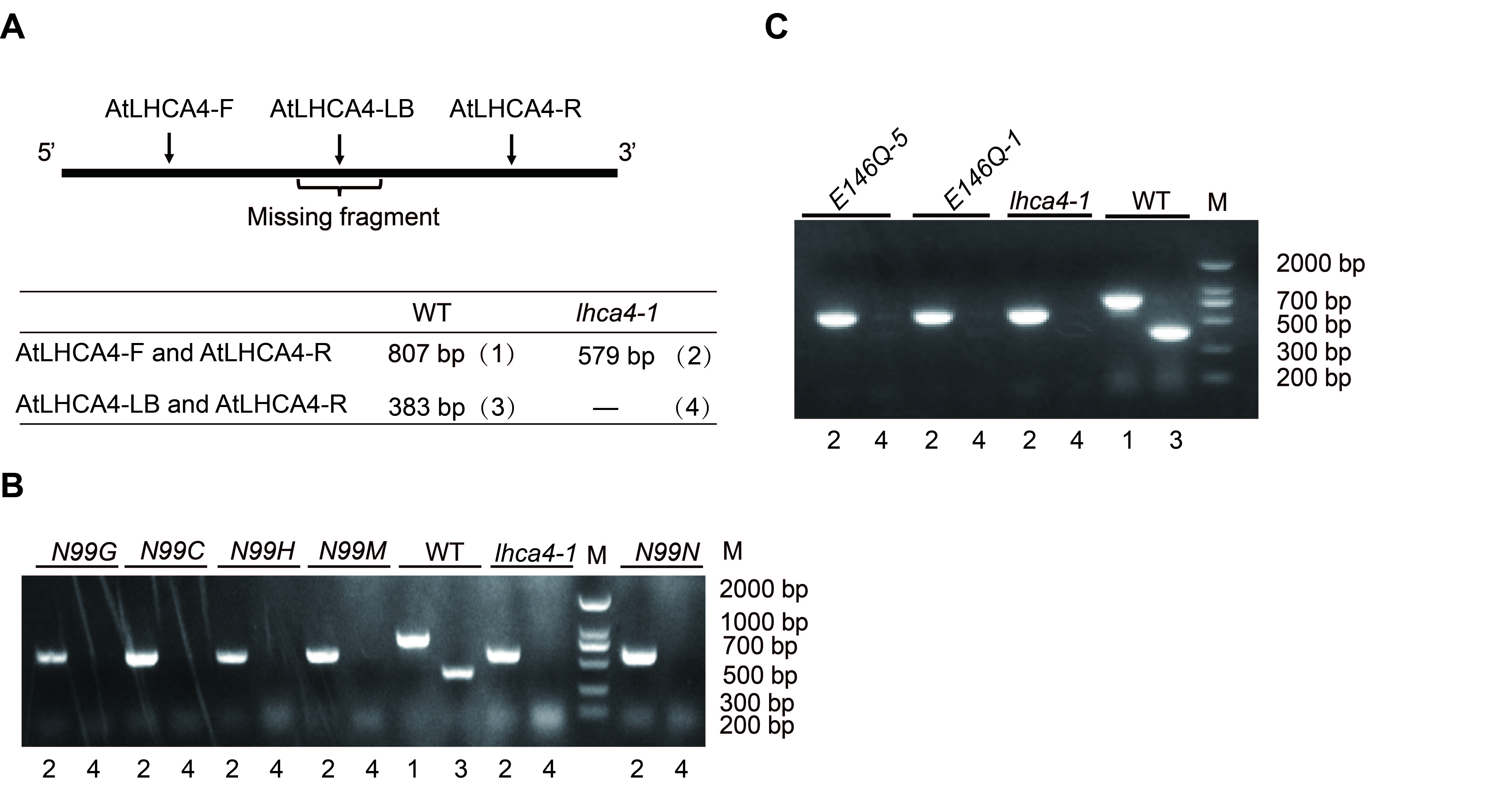


**Figure S3 Principle of primer design for mutant detection and detection of point mutant plants in the T1 generation.** (**A)** Principle of primer design for mutant detection. **(B-C)** Detection of point mutant plants of N99 (B) or E146 (C) in the T1 generation.


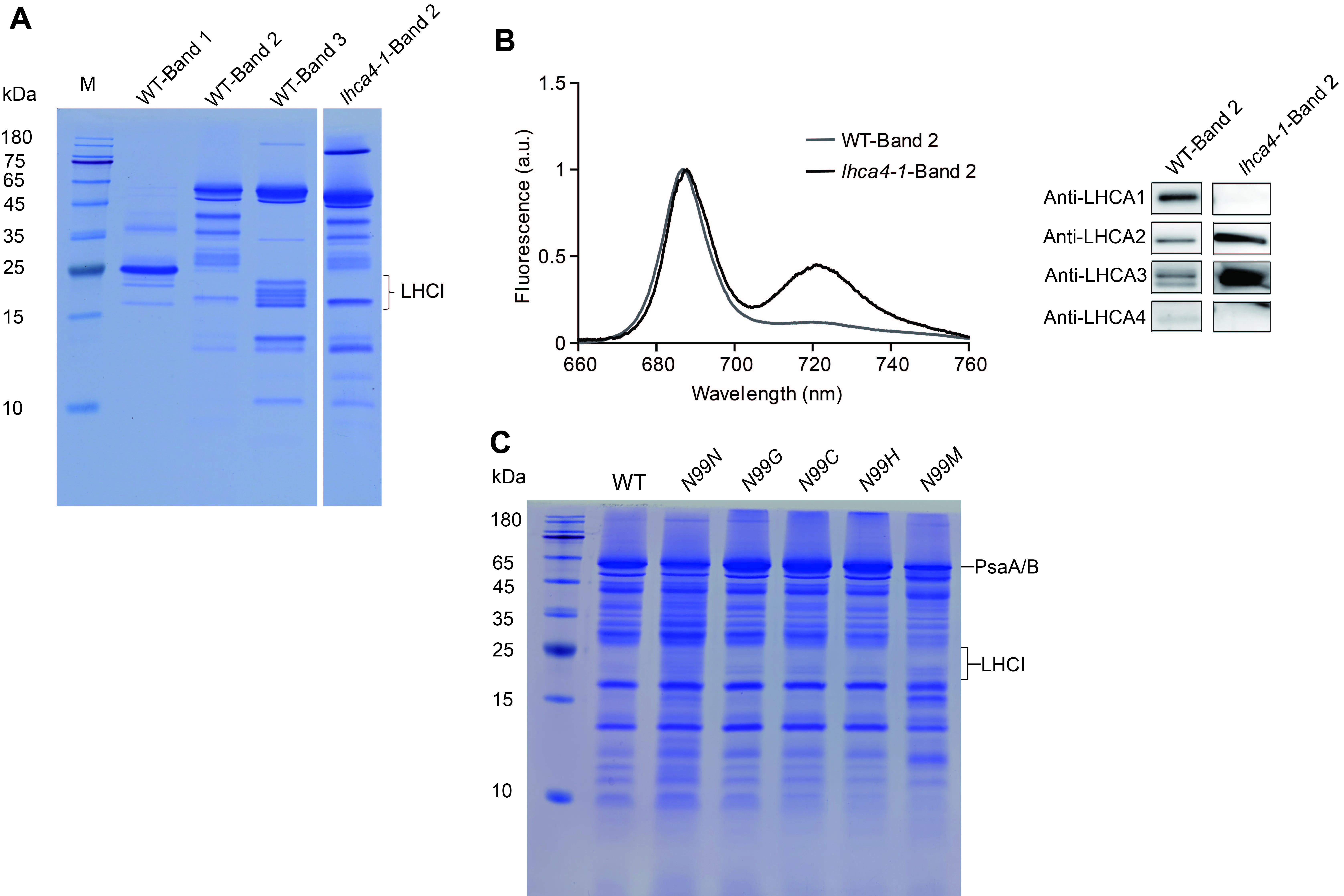


**Figure S4 Polypeptide composition of Band 2 from WT, *lhca4-1*, and N99 point mutant lines.**

**(A)** Polypeptide composition of Bands 1–3 from WT and Band 2 from *lhca4-1* as analyzed by Coomassie staining after SDS-PAGE. **(B)** Low-temperature (77K) fluorescence emission spectra and immunoblot analysis of Band 2 from the WT and *lhca4-1.* The excitation wavelength was 440 nm, and the spectra were normalized to their emission maxima (set to 1). Antibodies against LHCA1, LHCA2, LHCA3, and LHCA4 were used for the immunoblot analysis. **(C)** Polypeptide composition of Band 2 from N99 point mutant plants as analyzed by Coomassie staining after SDS-PAGE (samples were loaded onto SDS-PAGE at 2.5 μg Chl per lane in A, B and C.)

**Table S1. Primers used in this study**

| **Primer name** | **Sequence (5′ to 3′)** | **Function** |
| --- | --- | --- |
| Target 1 | CCGCGACTTGTCGTTTACATCGA | The target sequence for Cas9 editing system in LHCA4 genome |
| Target 2 | GCATCGCCTGATTATCTCACCGG | The target sequence for Cas9 editing system in LHCA4 genome |
| AtLHCA4-F | CCTCCGTCAAATCAACCACG | Cloning *LHCA4* gene segment, pairing with AtLHCA4-R |
| AtLHCA4-R | CTTGGTGAAAACTTCCGGCA | Cloning *LHCA4* gene segment, pairing with AtLHCA4-F/LB |
| AtLHCA4-LB | CTCACCGGCAGGTAATGTTT | Missing gene fragment in *lhca4-1*, pairing with AtLHCA4-R |
| LHCA4 F | AAATGTTCCTGAGTGGTACGAT | Real-time quantitative primer with *LHCA4*, pairing with LHCA4R |
| LHCA4 R | GGTTAAAGATTCCACCAGGGTA | Real-time quantitative primer with *LHCA4*, pairing with LHCA4F |
| SAND F | AACTCTATGCAGCATTTGATCCACT | Real-time quantitative primer with *SAND*, pairing with SAND R |
| SAND R | TGATTGCATATCTTTATCGCCATC | Real-time quantitative primer with *SAND*, pairing with SAND L |
| LHCA4-GL | GCAGAGCTGGTCGGCGGACGATGGGCTATG | Cloning *LHCA4* CDS with G99 substitution N99, pairing with LHCA4-GR |
| LHCA4-GR | CATAGCCCATCGTCCGCCGACCAGCTCTGC | Cloning *LHCA4* CDS with G99 substitution N99, pairing with LHCA4-GL |
| LHCA4-CL | GCAGAGCTGGTCTGCGGACGATGGGCTATG | Cloning *LHCA4* CDS with C99 substitution N99, pairing with LHCA4-CR |
| LHCA4-CR | CATAGCCCATCGTCCGCAGACCAGCTCTGC | Cloning *LHCA4* CDS with C99 substitution N99, pairing with LHCA4-CL |
| LHCA4-HL | GCAGAGCTGGTCCACGGACGATGGGCTATG | Cloning *LHCA4* CDS with H99 substitution N99, pairing with LHCA4-CR |
| LHCA4-HR | CATAGCCCATCGTCCGTGGACCAGCTCTGC | Cloning *LHCA4* CDS with H99 substitution N99, pairing with LHCA4-CL |
| LHCA4-ML | GCAGAGCTGGTCATGGGACGATGGGCTATG | Cloning *LHCA4* CDS with M99 substitution N99, pairing with LHCA4-MR |
| LHCA4-MR | CATAGCCCATCGTCCCATGACCAGCTCTGC | Cloning *LHCA4* CDS with M99 substitution N99, pairing with LHCA4-ML |
| LHCA4-QL | TTGTTCGTGATCCAGTTCATATTGTTTCAT | Cloning *LHCA4* CDS with Q146 substitution E146, pairing with LHCA4-QR |
| LHCA4-QR | ATGAAACAATATGAACTGGATCACGAACAA | Cloning *LHCA4* CDS with Q146 substitution E146, pairing with LHCA4-QL |
| LHCA4L | CAGGTCGACTCTAGAGGATCCATGGCTACTGTCACTACTCATGCC | Cloning *LHCA4* overall CDS |
| LHCA4R | GGGAAATTCGAGCTCGGTACCTTAGTTGAAGGTTTGGACAATAGTGTT | Cloning *LHCA4* overall CDS |

**Table S2 Protein stability prediction**

| PDB_file | Chain | Wild_res | Res_pos | Mut_res | Pred_ddg |
| --- | --- | --- | --- | --- | --- |
| 4xk8.pdb | 3 | N | 99 | A | –0.628 |
| 4xk8.pdb | 3 | N | 99 | V | –0.206 |
| 4xk8.pdb | 3 | N | 99 | L | –0.037 |
| 4xk8.pdb | 3 | N | 99 | G | –0.878 |
| 4xk8.pdb | 3 | N | 99 | S | –0.431 |
| 4xk8.pdb | 3 | N | 99 | W | –0.663 |
| 4xk8.pdb | 3 | N | 99 | T | –0.245 |
| 4xk8.pdb | 3 | N | 99 | Q | –0.572 |
| 4xk8.pdb | 3 | N | 99 | E | –0.051 |
| 4xk8.pdb | 3 | N | 99 | C | 0.321 |
| 4xk8.pdb | 3 | N | 99 | R | –0.037 |
| 4xk8.pdb | 3 | N | 99 | P | –0.206 |
| 4xk8.pdb | 3 | N | 99 | D | –0.065 |
| 4xk8.pdb | 3 | N | 99 | F | –0.791 |
| 4xk8.pdb | 3 | N | 99 | I | –0.037 |
| 4xk8.pdb | 3 | N | 99 | H | –0.891 |
| 4xk8.pdb | 3 | N | 99 | M | 0.512 |
| 4xk8.pdb | 3 | N | 99 | Y | –0.435 |
| 4xk8.pdb | 3 | N | 99 | K | –0.029 |

Amino acid residue 99 was replaced with all other possible amino acids, and the stability value of the resulting protein was calculated with Rosetta software. The amino acids returning the highest (C/M) or lowest (G/H) values were selected for analysis.

**Table S3 Photodensity of sucrose density gradient ultracentrifugation**

| **Genotype** | **Band 2 (photodensity)** | **Band 3 (photodensity)** |
| --- | --- | --- |
| WT | 1 | 1 |
| *lhca4-1* | 2.08 | — |
| *N99N* | 1.49 | 0.98 |
| *N99G* | 1.62 | 0.80 |
| *N99C* | 1.21 | 0.80 |
| *N99H* | 1.24 | 0.81 |
| *N99M* | 1.33 | 0.84 |

**Table S4 FWHMs of 77K fluorescence emission spectra of plants with single amino acid substitutions at N99 in LHCA4 and PSI-LHCI isolated from these plants.**

| **Materials** | | **FWHM** |
| --- | --- | --- |
| WT | 30.0±0.3 nm | |
| *lhca4*-1 | 30.3±0.4 nm | |
| *N99N* | 30.5±0.4 nm | |
| *N99G* | 30.9±0.4 nm | |
| *N99C* | 29.8±0.6 nm | |
| *N99H* | 29.9±0.4 nm | |
| *N99M* | 31.1±0.7 nm | |
| PSI-LHCI@WT | 31.3±0.3 nm | |
| PSI-LHCI@*N99N* | 31.0±0.5 nm | |
| PSI-LHCI@*N99G* | 30.0±0.1 nm | |
| PSI-LHCI@*N99C* | 30.5±0.1 nm | |
| PSI-LHCI@*N99H* | 30.2±0.2 nm | |
| PSI-LHCI@*N99M* | 32.5±0.3 nm | |
